# Is the use of contraceptives associated with periodontal diseases? A systematic review and meta-analyses

**DOI:** 10.1186/s12905-021-01180-0

**Published:** 2021-02-01

**Authors:** Micaele Maria Lopes Castro, Maria Karolina Martins Ferreira, Iasmin Encaua Essashika Prazeres, Paula Beatriz de Oliveira Nunes, Marcela Baraúna Magno, Cassiano Kuchenbecker Rösing, Lucianne Cople Maia, Rafael Rodrigues Lima

**Affiliations:** 1 Laboratory of Functional and Structural Biology, Institute of Biological Sciences, Federal University of Pará, Augusto Corrêa street, n 1, Guamá, Belém, PA 66075-110 Brazil; 2 Department of Pediatric Dentistry and Orthodontics, School of Dentistry, Federal University of Rio de Janeiro, Rio de Janeiro, Brazil; 3 Department of Periodontology, Faculty of Dentistry, Federal University of Rio Grande do Sul, Porto Alegre, Brazil

**Keywords:** Hormonal contraceptives, Periodontal disease, Periodontium, Systematic review

## Abstract

**Background:**

Previous studies indicated an impact of hormonal contraceptive use on oral health. This systematic review aimed to investigate the evidence supporting the impact of the use of hormonal contraceptives and periodontal diseases.

**Methods:**

This study is based on Preferred Reporting Items for Systematic Reviews and Meta-Analyses (PRISMA) and based on the PECO acrostic. Inclusion criteria comprised observational studies including women (P), which evaluated hormonal contraceptive users (E) and hormonal contraceptive non-users (C), to verify the association between this hormonal therapy and the periodontal diseases (O). Searches were performed on 5 databases: PubMed, Scopus, Web of Science, Cochrane Library, LILACS and grey literature (OpenGrey and Google Scholar). After the selection process, the included studies were evaluated qualitatively. Moreover, quantitative data were analyzed in two meta-analyses for clinical attachment loss (CAL) and probing depth (PD). Finally, the level of certainty was measured with the GRADE (Grading of Recommendations Assessment, Development, and Evaluation) tool between periodontal clinical parameters.

**Results:**

18 articles were eligible for the qualitative synthesis and 7 of them were selected for quantitative analysis. Hence, 15 of the eligible articles reported an association between the use of hormonal contraceptives and severity of periodontal disease. However, 6 articles demonstrated high risk of bias and were excluded from quantitative synthesis. The meta-analysis showed a statistically significant difference for CAL (MD 0.24 [0.09, 0.40]; *p* = 0.002), but in PD (MD 0.05 [− 0.05, 0.15]; *p* = 0.33) such difference was not identified. A very low level of evidence was found between the clinical parameters.

**Conclusions:**

The use of hormonal contraceptives may be associated to severity of periodontal diseases. However, the quantitative analysis points to an inconclusive outcome due to the high level of heterogeneity. The association is biologically plausible, however additional studies are warranted to better elucidate the clinical significance of this possible association.

## Background

Hormonal contraceptives are drugs used to prevent pregnancy that can also be used in specific situations as family planning, menstrual cycle regularization, reduction in the incidence of ovarian cysts, etc. [1]. Among the hormonal contraceptive alternatives, there are many birth control options including contraceptive pills, contraceptive patches, implants, injections, intravaginal, and intrauterine delivery. Generally, in the composition of each contraceptive drug, there are two synthetic hormones, estrogens and progestins, which act by performing selective inhibition of pituitary function [1, 2].

This mechanism of action generates the pituitary secretion inhibition of luteinizing hormone and follicle stimulating hormone, resulting in the hindrance of ovum release by ovary, promoting contraception [3]. However, despite the benefits, the use of hormonal contraceptives, mainly orally, is associated with systemic adverse effects, as thromboembolic and cardiovascular complications [4].

Periodontal disease in the initial phase is restricted to gingival tissues. On the other hand, in an advanced phase, this condition affects periodontal support tissues, and is called periodontitis [5]. For diagnosis of periodontal diseases, clinical measurements are used with several standardized indexes by the scientific literature which has the goal to reflect the etiology and pathogenesis of periodontal disease [6].

In the oral cavity, this relationship has been associated with periodontal status, because the sexual steroids play significant roles in modulating inflammatory response of periodontal tissues and may alter the response to oral these structures during different phases of life, including puberty, menstruation, pregnancy, menopause and postmenopausal [7–10].

Although there is a systematic review published with a similar scope [11], doubts still persist regarding the methodological quality of the published articles, as well as an integrated analysis by a meta-analysis combining the results of the selected articles. In addition, the analysis of the certainty of evidence which enables guidance on clinical decision-making process.

In this context, the present systematic review aims to investigate the scientific evidence that supports the clinical observations related to the association between the use of hormonal contraceptives and periodontal diseases.

## Methods

### Protocol and register

This systematic review was registered on PROSPERO under the code CRD42018115606 and was developed according to Preferred Reporting Items for Systematic Reviews and Meta-Analyses (PRISMA) and the Cochrane Protocol for systematic reviews (Additional file 1: Table S1). [12].

### Focused question and selection criteria

To perform this review, the following focused question was raised: “Is there an association between the use of hormonal contraceptives and periodontal diseases?”. To answer this focused question, the PECO strategy was used: observational studies in adult women (P), hormonal contraceptive users (E) and non-hormonal contraceptive users (C) that were evaluated to identify the presence or absence of the association between the use of hormonal contraceptives and outcomes related to periodontal diseases (O). Pilot studies, case reports, descriptive studies, review articles, opinion articles, technique articles and guidelines, studies investigating the use of barrier contraceptives, and studies which do not report on the clinical parameters of periodontitis were discarded.

### Search strategy

Searches were performed on the following electronic databases: Pubmed, Scopus, Web of Science, LILACS and Cochrane Library. Google Scholar and The Open Grey were used as gray literature sources. No restriction of year or language were applied. The search strategy was composed by MESH and entry terms and adapted according to each database, using boolean operators (OR, AND) to combine the searches. The MeSH terms used included “Contraceptives, oral, hormonal” or “Contraceptive Agents” or “Contraceptive Agents, Female” or “Contraceptives, Oral” or “Contraceptives, Oral, Sequential” or “Vaccines, Contraceptives” or “Reproductive Control Agents” and “Periodontal Diseases” or “Gingivits” or “Periodontium” or “Gingiva” or “Alveolar Process” or “Periodontitis” or “Chronic Periodontitis” or “Periodontal Attachment Loss” or “Alveolar Bone Loss” or “Oral Health” (Additional file 2: Table S2).

Manual search was also performed. It is an important complementary step to find possible eligible studies that may not have been recovered in the search strategy. This step was carried out in two ways, during the search in the bibliographic references of each selected study or in classic literature reviews and systematic reviews previously published with similar themes.

The searches were performed until December 2019. Although, a search alert was created in each database to notify new studies according to the outlined search strategy. After the searches, the citations found in each database were exported to a reference manager (EndNote^®^, version X7, Thomson Reuters, Philadelphia, EUA). Articles indexed in more than one database were considered only once.

All evaluations, including the searches, studies selection, risk of bias evaluation, data extraction was performed in pairs, independently, by two examiners (MMLC and PBON). After each analysis, MMLC and PBON met to discuss the encountered data. Any disagreement between the examiners were resolved by a third reviewer (MKMF).

### Studies selection process

After the importation to a reference manager, the duplicated results were removed (EndNote^®^, versão X7, Thomson Reuters), both automatically and by manual review. Subsequently, the articles were excluded by titles and abstracts, and after for full-text reading according to the PECO’s strategy within the eligibility criteria.

Moreover, the lists of references from each included article were also researched manually in order to find additional studies that could be included in the review.

### Data extraction

For each selected manuscript, the following information was collected: author, country, publication year, study design, age, sample size, type of hormonal contraceptive, statistical analysis and main results. The authors of the studies were contacted if relevant data were absent in the articles.

### Quality analysis and risk of bias

The guidelines proposed by Fowkes and Fulton [13] were used used in this systematic review to evaluate the quality and risk of bias of the included studies in order to verify wheter the methods and research results were sufficiently valid to produce useful information [13].

### Quantitative analysis (meta-analysis)

The data of each study included in quantitative synthesis were analyzed in Review Manager (versão 5.2) to evaluate the association between the use of hormonal contraceptives and the presence of periodontal diseases.

The studies that reported results using the same methods were intended to meta-analyses. Then, the mean difference (MD), with a confidence interval of 95% (IC) was calculated. Only studies with low risk of bias were included in the meta-analysis. If any information needed for the meta-analysis was missing from any of the selected studies, the authors were contacted to provide the missing data. [14].

The heterogeneity was tested by the I^2^ index and, if possible, sensitivity analyzes were performed to estimate and verify the influence of the studies, one by one, on the subgroup and pooled results, when the heterogeneity was substantial or considerable (50–100%) [15]. Two periodontal clinical parameters were included in these meta-analyses: CAL and PD. These clinical parameters allow assessing the level clinical inflammation as a result of periodontal disease (PD) as well as the changes related to the supporting periodontal tissues (CAL) [16]. Currently, those parameters have been established as the gold-standard for diagnostic of patients with periodontal diseases [17].

### Level of evidence: grading of recommendations assessment, development, and evaluation (GRADE)

To evaluate the certainty of evidence among the studies, the GRADE tool [18] was applied using the following periodontal parameters: CAL and PD. The included articles were evaluated according to study design, risk of bias, inconsistency, indirectness and imprecision.

The certainty of the evidence (certainty in the estimates of effect) was determined for the outcome using the Grading of Recommendations Assessment, Development and Evaluation (GRADE) approach [18].

## Results

### Included studies

A total of 1231 articles were identified through the search for databases and grey literature. Then, 531 articles were excluded after duplicates removal, resulting in 700 articles. Among them, 620 were excluded after reading the titles.

**Fig. 1:**
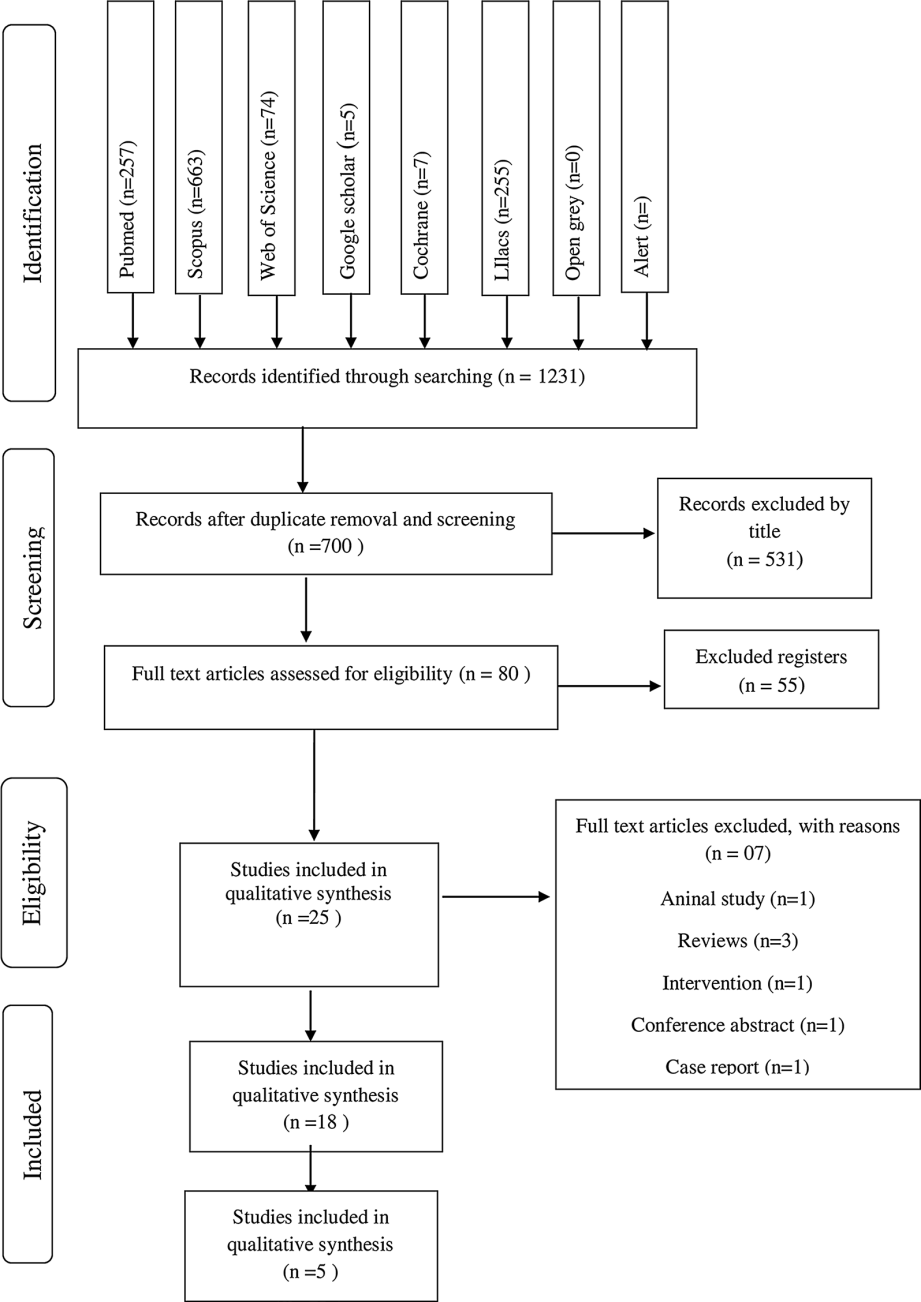
Flowchart of the selection strategy of studies according to PRISMA protocol

From these remaining articles, 80 articles were excluded after their abstract reading, and 55 studies were eliminated, the remaining 25 articles for full-text reading to be evaluated according to eligibility criteria. Finally, 7 were excluded for the following reasons: animal study (1), review (3), interventional study (1), conference summary (1) and case report (1). Thereby, 18 articles met the inclusion criteria and were selected for qualitative synthesis [19–36]. From these, 5 articles were directed to the quantitative analysis [19, 24, 31, 32, 36] (Fig. 1).

### Description of study characteristics

According to study design, fifteen are classified as case–control studies [19–25, 27–29, 31–34, 36] and three as cross-sectional [26, 30, 35]. The type of hormonal contraception mostly used was oral administration (present in fifteen studies). Seck et al. [34] included three types of hormonal contraception, examining patientes that used oral (Lo-Femenal^®^), injectables (Depo-Provera^®^), and implants (Norplant^®^). The study of Tilakaratne et al. [36] analyzed the injectable and oral contraceptive users, while Kazerooni et al. [30] evaluated only implant contraceptives of Levonorgestrel. All the studies performed clinical analyses of periodontal condition (Table 1).

**Table 1:** Domains and risk of bias according to Fowkes and Fulton

Guidelines	Checklist	Description
Study design appropriate to objectives?	Objective common design	The type of study was marked in the appropriate type of study. If the type of study was appropriate according to the study design, it was labelled as "0", and as "++" if it was not appropriate
	Prevalence Cross-sectional	
	Prognosis Cohort	
	Treatment Controlled trial	
	Cause Cohort, case–control, cross-sectional	
Study sample representative?	Source of sample	The domain was considered [0] in cases of detailed origin, [+] to a specified origin of only one group and [++] in cases of absence of specification of the source of the groups
	Sampling method	The item was assigned [0] for a full description of sampling method, [+] for poor or no explanation of sample method, with no problem in matching between groups, and [++] for poor or no description of sample method, interfering in the matching of the groups
	Sample size	A minor problem [+] was considered when the sample was not representative or did not report a sample calculation. To a major problem, [++] was considered when no sample calculation was provided, and the number of participants was less than 50 participants, [0] was considered in the absence of the above factors
	Entry criteria/exclusion	A minor problem [+] was attributed when the control and case group reported current use of antibiotics or anti-inflammatories, diabetes, smoking or pregnancy. In the case of presence of more than two previously mentioned items, it was considered as a major problem [++]
	Non-respondents	The [0] was attributed when there was no refusal to participate in the study, [+] was assigned when there was the refusal, but did not compromise the sample, and [++] when there were refusal and impairment of the sample size
Control group acceptable?	Definition of controls	It was attributed [0] when all characteristics of the control group were described, [+] when any information was pendent as the origin of the control group, the selection criteria and a different origin between case and control groups and [++] when two or more items described in previously items
	Source of controls	It was considered [0] when the control group was referred, [+] when the origin of groups was different, but with reasons and [++] when the groups presented different origins without reasons
	Matching/randomization	In this item, [0] was assigned to cases of randomized/matched groups, [+] to cases of no description of randomization, but with a matching of groups and [++] to no explanation of randomization or matching
	Comparable characteristics	It was attributed [0] to matched groups or not matched by the impossibility of being subsequently adjusted and [++] the presence of unpaired variables that were not paired or adjusted
Quality of measurements and outcomes?	Validity	It was considered [0] when the evaluation method applied is appropriate; [+] when using a single method, but with appropriate sensitivity with good specificity; [++] when using a single method, without an adequate specificity or good sensitivity
	Reproducibility	It was considered [0] whether the evaluation methods were well described; [+] when a lack description of any step of the method was presented, for example, the identification of the patients of the groups studied in laboratory samples, evaluations at different times or application of various methods between groups of individual pathology; [++] when two or more of the previous items are present
	Blindness	The condition of the study participants was considered to be "Blind," in this case being assigned the signal [0], in cases of "not blind" the signal [++] was attributed
	Quality control	It was considered a problem when the examiner was not qualified; a partial periodontal exam was performed [not in all teeth or not in all the six periodontal sites/teeth], the measurement of periodontitis was only radiographic or the absence of the number of evaluated teeth sites. A Minor problem [+] was considered when 2 of these characteristics were present, and a major problem [++] if more than 2 of these characteristics were present
Completeness	Compliance	It was assigned [0] for a sample size that remains the same from the beginning to the end or decreases without compromising the power of the test; [+] for differences in sample size at the end of the study, compromising the power of the test, but with reasons and adjusts; [++] for difference in sample size at the end of the study, compromising the power of the test, without reasons
	Dropouts	The [0] was scored when there is no loss during the study, [+] when there is a withdrawal that involves the inclusion criteria, such as age, sex, [++] when there is withdrawal and it compromises more than one criterion
	Deaths	This item was scored as Not Applicable [NA], due to the type of PECO strategy
	Missing data	In this item, [0] was assigned to cases of randomized/matched groups, [+] to cases of no description of randomization, but with a matching of groups and [++] to no description of randomization or matching
Distorting influences?	Extraneous treatments	In this item, [0] was considered when there were no external influences; [+] when there are external influences, but that does not interfere in the results; [++] when there are external influences and interferes with the results
	Contamination	This item was scored as Not Applicable [NA], due to the type of PECO strategy
	Changes over time	In this item, [0] was attributed to data collected in the same period; [+] to data obtained from the control group and the study group at different times that may cause distortions; [++] when the previous item was associated with data from studies already published
	Confounding factors	A problem was assigned when the data analysis involved enrollment of persons < 5 years. Menopausal woman, smokers, diabetics and obese. A minor problem [+] was assigned when 1 or 2 of these characteristics were present and a major problem [++] if there were 3 or more
	Distortion reduced by analysis	It was considered [0] when it cites the adjustments of the covariates that present distortions; [+] when the article report adjustment, but does not say the criteria; [++] when distortion was identified, without adjustment
Summary questions	Bias: Are the results erroneously biased in a certain direction?	YES or "NO" answers were assigned to each question. If the answer is NO to the three questions, the article is considered reliable, with low risk of bias
	Confounding: Are there any serious confusing or other distorting influences?	
	Chance: Is it likely that the results occurred by chance?	

### Quality assestment and risk of bias

Furthermore, after a detailed evaluation of methods and results, the studies were analyzed to verify the possibility of “biased results”, “confouding” and “occurrence by chance” (Table 2). Four studies were classified with high risk of bias [23, 27, 28, 30]. The major problems observed were in relation to the sample (sample size and definition of inclusion and exclusion criteria) and the acceptability of the control group (absence of description of the randomization/correspondence process).

**Table 2:** Characteristics of included studies

Author, year, country;	Participants			Age	Periodontal Evaluation	Administration	Statitic analysis	Results
	Type of study	Study site	Sample size					
Smadi and Zakarya [20]; Jordan	Case Control	Ginecology Clinic at the Islamic Hospital	(281) Case Group (142) Control Group (139)	Mean: Case Group (26.7 ± 7.5) Control Group (24.5 ± 6.9)	OHI-S, SBI, CAL, GI (number of teeth examined)	Oral	Student’s *t*‑test	There was statistically significant difference in all evaluated clinical parameters among groups OHI-S (*p* = 0.002), GI (*p* = 0.001), SBI (*p* = 0.001), CAL (*p* = 003)
Farhad et al. [19]. Iran	Case control	Azad Dental School of Khorasgan	(60) Case group (35) Case Control (25)	Mean: 28.5 years	PD, CAL, PI, BOP	Oral	Student’s t-test and Mann–Whitney test	Only PI did not show statistically significant difference among groups (*p* < 0.05)
Abd-Ali [21]	Case Control	Hospital of collage of Al-Mustansiri	(80) Case group (40) Control group (40)	Mean ± SD Case Group 29.15 ± 5.83 Control Group 28.45 ± 3.75	GI (GI of Loe and Silness (39)) Niveis de IgA	Oral	Simple linear regression	Gingival index was significantly higher among oral contraceptive users than non-users (*p* < 0.01)
Domingues; Ferraz. et al. [23]; Brazil	Case Control	Bauru School of Dentistry, Universaty of São Paulo, Brazil	(50) Case Group (25) Control Group (25)	Mean: 24 years	PD, CAL, SBI and Pl.I	Oral	unpaired *t,* Pearson’s correlation test, Spearman’s correlation test for non-linear measurements	The test group showed increased PD (*p* < 0.0001) and SBI (*p* < 0.0001) as compared to controls. No significant differences between groups were found in CAL (*p* = 0.11). The control group showed greater Pl.I than the test group (*p* < 0.0001)
Brusca et al. [22]; Argentina	Case Control	University and private clinic offices	(92) Case Group (41) Control group (51)	Mean ± SD 30.34 ± 6.2 Years	GI, PI, PD, CAL	Oral	Chi-square test	Contraceptive users, particularly smokers, demonstrated a statistically significant higher prevalence of severe periodontitis (*p* < 0.01) and deeper probing depths ≥ 5 mm) than non-users. Moreover, contraceptive users presented higher GI scores and CAL, ≥ 2 and, ≥ 5 mm, respectively, than non-users (*p* < 0.01)
Haerian-Ardakan et al. [24]; Iran	Case Control	Kasturba Medical College	(60) Case group (35) Control Group (25)	Mean: 24 years	PD, BOP, PI, CAL	Oral	Mann–Whitney and t-test, *p* < 0.05	A statistically significant difference in GI and BOP was observed (*p* < 0.0001)
Vijay et al. [24], Índia	Case Control	Kasturba Medical College	(65) Case group (43) Control group (22)	Mean ± SD Case Group I: 26.94 ± 3.28 II: 29.05 ± 2.04 III: 30.75 ± 2.06 Control Group: 25.09 ± 3.26	PI (Silness e Loe) PI (Russel), Radiographic paramaters	Oral	Student’s t’s test	Statistically significant difference was observed in all parameters (*p* < 000.1)
Kazerooni et al. [30]; Iran	Cross-sectional		(148) Case Group (101) Control group (47)		PD	Levonorgestrel Implants	Mann–Whitney test, test V2	In the study group, the pocket depth around the distal (*p* = 0.001) and middle (*p* = 0.001) aspects of the anterior teeth and the middle aspect of the premolars (*p* = 0.02) was significantly increased at 6 months. In comparison with the control group, except around the distal aspect of the premolars (*p* = 0.09) and the mesial aspect of the anterior teeth (*p* = 0.07), pocket depth was significantly increased in the study group
Seck et al. [34]; França	Case Control	Centre de Soins Integres Abass Ndao	(200) Case Group (100) Control Group (100)	15–45 years	PI (Loe and Silness), GI (Loe & Silness), PD, CAL	Oral, Injectable, implants	Wilcoxon test	With equal hygiene, the scores of the gingival index were significantly higher among women under contraceptive (*p* < 0.001). Inflammation was significantly more marked for the women Who used contraception in injectable form compared to the control group (*p* < 0.001). Probing depth and clinical attachment loss were significantly higher among women under contraceptive (*p* < 0.001)
Mullualy et al. [32]; Irlanda do Norte	Case Control	University Belfast, Belfast, Northern Irland	(42) Case group (21) Control Group (21)	Mean: 26.5 years	GI (GI of Loe and Siiness (39)), PI, BOP, PD, CAL	Oral	ANOVA Regression analysis	Current pill users had deeper mean probing depths compared to nonusers (*p* = 0.006) and more severe attachment loss *p* = 0.015). Pill users had more sites with bleeding on probing (*p* = 0.017)
Taichman et al. [35]	Cross-sectional	First and third National Health and Nutrtion Examination Suryeys (NHANES I and NHANES III)	NHANES I: (4413) Case Group: (3307) NHANES II: 4169 Case group: (842) Control Group: (3327)	Mean: 33.5 years	PI, OHI-S, CAL, PD	Oral	Logistic regression multivariable	Premenopausal women who use HC are more likely to present severe gingivitis and periodontitis (*p* < 0.05). However, in the postmenopausal phase it was reported that estrogen supplementation would be associated with improved gingival health
Tilakaratne et al. [36]; London	Case Control	University Of Peradeniya. Sri-Lanka;	(88) Case group (49) Control group (39)	Mean: 26.5 years	PI, GI, CAL	Oral and injetable	one-way ANOVA	The contraceptive users had a significantly higher level of GI and CAL, compared to the non-users (*p*, 0.001)
Jensen et al. [28]; EUA	Case control	University of Minnesota Obstetrics and Gynecological Clinic	(104) Case group-NPP (50) Control Group- NP: (54)	Mean: 29 years	GI,GCF, DP	Oral	Kornman and Loesche	There was not statistical difference among groups in all parameters (p > 0.05)
Pankhurst et al. [33]; London	Case control	Margaret Pyke Centre London	(151) Case group (112) Control group (39)	Mean: 29 years	% PI, GI, CAL, PD	Oral	Two-way analysis of variance	A statistically significant higher gingival inflammation was observed and related to the duration of drug therapy. There were no significant differences in CAL between groups
Kalkawarf [29]; EUA	Case control	Dentustry clinic of the University of Nebraska College of Dentistry	(189) Case group (114) Control Group (75)	Mean: 26.5 Years	Mean Oral Debris Index an a mean Gingival Inflammatory Index	Oral	Mean ± SD	The use of different hormonal contraceptives is associated with different degrees of gingival inflammation depending on the concentration of progesterone and estrogen (*p* < 0.001)
Grower et al. [27]; EUA	Case control		(12) Case group (05) Control group (07)	Mean: 26.6 Years	PI, GI	Oral	Mean ± SD, Mann, Whitney U-test	There was not statistically significant difference among all parameters (p > 0.05)
Knight et al. [31]; Reino Unido	Case Control	Margaret Pyke Centre Of The Family in London	(171) Case group (89) Control group (72)	Mean: 17–23 years	PI, GI, CAL	Oral	Student’s t test	A statistically significant difference was observed only in GI (*p* < 000.1)
El-Ashiry et al. [26]; Egypt	Cross-sectional	University School of Dentistry, Cairo, Egypt	(120) Case group (70) Control group (50)	Mean: 28.5 years	Gengival Mean Calculus Scores	Oral	Gingival mean, Calculus Scores and T test	The highest effects of HC are observed during the first 3 months and gingival exudate only increases during the first 6 months, due to the greater release of mast cells during this period of HC intake (*p* < 0.01)

BOP: Bleeding on probing; CAL: Clinical attachment level; GCF: Gingival crevicular fluid; GI: Gingival index; OHI-S: Oral hygiene index; PD: Probing depth; PI: Plaque index; SBI: Sulcular bleeding index

In relation to the sample source, Domingues et al. [23] and Grower et al. [27] selected lower maximum age group making the sample unrepresentative, which was considered a major problem. In relation to the sampling method, Domingues et al. [23], Vijay et al. [24], Pankhurst et al. [33] and Grower et al. [27], didnot report how the sample selection was calculated/achieved, and this was considered a major problem. Farhard et al. [19], Domingues et al. [23], Mullally et al. [32], Tilakaratne et al. [36], Jensen et al. [28] and Grower et al. [27], did not perform the sample calculation and the samples were not enough to demonstrate a significant result. Vijay et al. [24], Kazerooni et al. [30], Jensen et al. [28] and Grower et al. [27], did not describe the exclusion criteria used in the selection of the study sample, which was considered a major problem. All included articles presented problem with blindness in the measurement of the outcomes. Knight et al. [31], Kazerooni et al. [30] and Grower et al. [27], did not mention confounding factors being considered a major problem. In summary, the quality assessment of all the included articles can be found on Table 3.

**Table 3:** Quality assessment according to Fowkes and Fulton [13]

Guideline	Verification list	Smadi et al. [20]	Farhard et al. [19]	Abd-Ali et al. [21]	Domingues et al. [23]	Brusca et al. [22]	Haerian-Ardakani Et al. [24]	Vijai et al. [24]	Kazerooni et al. [30]	Seck et al. [34]	Mullally et al. [32]	Tiachman et al. [35]	Tilakaratne et al. [36]	Jensen et al. [28]	Pankhurst et al. [33]	Kalkawarf et al. [29]	Grower et al. [27]	Knight et al. [31]	Elashiry et al. [26]
Study design appropriate to objectives?	Objective common design	−			−	−	−		−	−	−	−	−	−	−	−	−	−	−
	Prevalence Cross-sectional	−			−	−	−		−	−	−	0	−	−	−	−	−	−	−
	Prognosis Cohort	−			−	−	−		−		−	−	−	−	−	−	−	−	−
	Treatment controled trial	−			−	−	−		−	−	−	−	−	−	−	−	−	−	−
	Cause Cohort, case–control, cross-sectional	0	X	X	0	0	0		0	0	0	−	0	0	0	0	0	0	0
Study sample representative?	Source of sample	+	0	0	++	0	0	0	+	0	0	0	0	0	0	0	++	0	0
	Sampling method	+	0	0	++	+	+	++	+	+	+	0	+	+	++	+	++	+	++
	Sample size	+	++	0	++	+	+	+	+	+	++	0	++	++	+	0	++	+	0
	Entry criteria/exclusion	0	0	0	0	0	0	++	++	+	0	0	0	++	0	0	++	0	0
	Non-respondents	+	0	0	0	0	0	0	0	0	0	0	0	0	0	+	0	0	0
Control group acceptable?	Definition of controls	0	0	++	0	0	0	+	+	0	+	0	0	0	0	0	++	0	0
	Source of controls	0	++	0	0	0	0	0	0	+	0	0	0	0	0	0	++	0	0
	Matching/randomization	+	++	++	+	++	+	++	++	+	++	0	+	+	++	++	++	+	++
	Comparable characteristics	0	++	++	++	++	+	++	++	0	++	0	0	++	++	++	++	0	0
Quality of measurements and outcomes?	Validity	0	0	0	0	0	0	0	0	0	0	0	0	0	0	0	0	0	0
	Reproducibility	0	0	0	0	0	0	0	0	0	0	0	0	0	0	0	0	0	0
	Blinding	++	++	++	++	++	++	++	++	++	+	++	0	++	+	++	++	++	++
	Quality control	0	+	+	0	0	0	++	++	+	0	0	0	0	0	0	+	0	0
Completeness	Compliance	0	0	0	0	0	0	0	0	0	0	0	0	0	0	+	0	0	0
	Drop outs	0	0	0	0	0	0	0	0	0	0	+	0	0	0	0	0	0	0
	Deaths	NA	NA	NA	NA	NA	NA	NA	NA	NA	NA	NA	NA	NA	NA	NA	NA	NA	NA
	Missing data	0	0	0	0	0	0	0	0	0	0	+	0	0	+	++	++	0	+
Distortion influences?	Extraneous treatments	0	0	0	0	0	0	0	0	0	0	++	0	++	0	0	+	0	0
	Contamination	NA	NA	NA	NA	NA	NA	NA	NA	NA	NA	NA	NA	NA	NA	NA	NA	NA	NA
	Changes over time	NA	NA	NA	NA	NA	NA	NA	NA	NA	NA	NA	NA	NA	NA	NA	NA	NA	NA
	Confounding factors	+	0	0	+	0	0	+	++	−	+	+		++	0	+	+	++	0
	Distortion reduces by analysis	0	++		0	0	0	++	++	−	++	0	0	0	0	+	++	++	0
Summary questions	Bias: are the results erroneously biased in certain direction?	No	No	No	Yes	No	Yes	Yes	Yes	No	No	No	No	Yes	No	No	Yes	No	No
	Confusion: Are there any serious confusing or other distorting influences?	No	Yes	No	Yes	No	No	Yes	Yes	No	No	No	No	Yes	No	No	Yes	No	No
	Chances: Is it likely that the results occurred by chance?	No	No	No	Yes	No	No	No	Yes	No	No	No	No	Yes	No	No	Yes	No	No

0 = No problem; + = Minor problem; ++ = Major problem; NA = not applicable

### Meta-analysis and level of evidence

The meta-analyses results were presented separately for each periodontal parameter. The random effect model was used since the studies were not equivalent. This strategy had the objective to generalize the results of the meta-analyses.

**Fig. 2:**
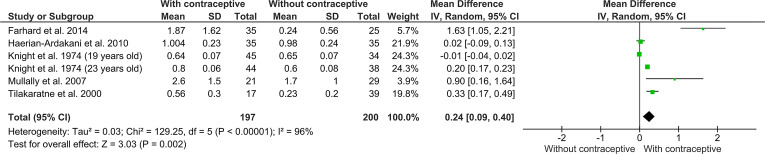
1st meta-analysis—clinical attachment loss

Five studies which evaluate CAL were included in this analysis [19, 24, 31, 32, 36]. Including all the articles, the heterogeneity was considerable (I^2^ = 96%). Contraceptive users (n = 197) had a mean CAL greater than the control group (n = 200) (MD 0.24 [0.09, 0.40]; *p* = 0.002) (Fig. 2) with very low certanty of evidence (Table 4). The inclusion of high risk of bias study did not change the effect significante (MD 0.15 [0.05, 0.24] *p* = 0.002).

**Table 4:** Level of evidence according to PD and CAL

Certainty assessment							No. of patients		Effect		Certainty	
№ of studies	Study design	Risk of bias	Inconsistency	Indirectness	Imprecision	Other considerations	With contraceptive	Without contraceptive	Relative (95% CI)	Absolute (95% CI)		
*CAL*												
5	Observational studies	Not serious	Serious^a^	Not serious	Serious^b^	None	197	200	–	MD 0.24 higher (0.09 higher to 0.4 higher)	⨁○○○ very low	
*PD*												
3	Observational studies	Serious^c^	Serious^a^	Not serious	Very serious^b,d^	None	91	89	–	MD 1.15 higher (0.29 lower to 2.59 higher)	⨁○○○ very low	

CI: confidence interval; MD: mean difference

^a^Considerable heterogeneity

^b^Total number of participants is lower than 400

^c^The inclusion of high risk of bias study change the effect significance

^d^Upper and lower CI is greater than 0.5

**Fig. 3:**
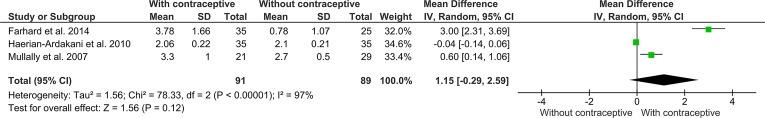
2nd meta-analysis—probing depth

Considering probing depth (PD), three studies were included in the analysis [19, 32, 36]. Including all articles, the heterogeneity was considerable (I^2^ = 97%). Contraceptive users (n = 91) had a mean PD greater than those who did not use it (n = 89) (MD 1.15 [− 0.29, 2.59]; *p* = 0.12) with very low certanty of evidence (Table 4). This data can be elucidated by the result of no difference between the groups in the study performed by Haerian-Ardakani et al. [24], which presented the greatest weight in our meta-analysis (Fig. 3). The inclusion of high risk of bias study change the significance of the effect (MD 0.56 [0.22, 0.91] *p* = 0.001).

## Discussion

The objective of this study was to evaluate the impact of the use of hormonal contraceptives on periodontal conditions. Then, a comparative analysis of users and non-users of hormonal contraceptives was performed. In this sense, a systematic review with meta-analyses was performed using important databases of literature in health sciences. The topic under study is of high clinical interest [37], however, the information presented in literature comes from studies that present some concerns in methodological features or low degree of evidence.

As from de board search, 18 articles were included in this systematic review without restrictions for the type of hormonal contraceptives. Concerning the included studies, 15 pointed worse periodontal clinical parameters in contraceptive users [19–26, 29, 30, 32–36]. However, the quantitative analysis indicates a potentially inconclusive outcome due to the heterogeneity detected in the articles, also highlighted in the level of evidence analysis, consequently compromising the evaluation of this association with the possibility of a more in-depth risk approach.

In order to formulate the scientific research question, our systematic review used the PECO’s strategy [38], which allows evaluating a potential risk or prognosis of contraceptive users to periodontal diseases, conditions characterized by the existence of a pathological inflammatory process in the periodontal structures (gums, cementum, periodontal ligament, and alveolar bone). Based on these cited premises, this systematic review and meta-analysis evaluates observational studies, mainly with cross-sectional design and case–control. Then, the results have the potential to generate information about greater or lesser odds of women using hormonal contraceptives present clinical manifestations of periodontal diseases. From this type of information, policies for the prevention of periodontal diseases could be proposed, both at the collective level (woman’s health) and in a personalized dentistry approach.

All the included studies collected data from anamnesis and clinical measures as, for example, OHI-S, CAL, gingival index (GI), PD, plaque index (PI), bleeding on probing (BOP), Mean Debris Index, Gingival Mean and Calculus scores. Other studies performed radiographic complementary analysis [25] and laboratory analysis of gingival crevicular fluid [28]. These analyses allow not only evaluation of the inflammatory status of periodontal tissues (GI, PI, BOP, Gingival Mean, Calculus Scores) [39, 40], but also the analysis of loss of periodontal tissue support (PD, CAL) [16], and need for periodontal treatment (PI, OHI-S) [41]. However, it is important to emphasize that the wide range of the collected clinical parameters prevents data from being pooled and more specific conclusions drawn. Additionally, some parameters analyzed presented limitations according to the contemporary knowledge, for example, Russel’s index and OHI-S.

Regarding the hormonal therapies with steroids and progesterone, most of them were hormonal oral administration, following by implanted devices and injectable contraceptives. Similarly to periodontal parameters, it is important to describe that hormonal contraceptives are quite variable. It is recognized in the contraception field, the evolution of the methods also has modified the prescriptions, and probably their adverse effects. It is known that efforts have been made to improve the effectiveness of contraception, with the reduction of hormonal load. This trend has the potential to minimize the adverse effects related to periodontal disease manifestations, especially in the function of the hormonal dosage, the probable cause of the adverse effect presented in this review.

Sex hormones are considered modifying factors, they are able to modulate the inflammatory response of tissues, including periodontal tissues. Studies indicate that gingival keratinocytes, gingival fibroblasts presented in the periodontal ligament, and in the lamina propria have receptors for sex hormones, such as estrogen and progesterone [42, 43]. Thus, steroid hormones are able to indirectly modulates periodontal tissue. Estrogen is able to modulate collagen metabolism and angiogenesis, in addition to promoting an increase in tissue glycogen production and reducing keratinization of the gingival epithelium, causing a reduction in the epithelial barrier. In addition, it modifies cell proliferation such as increased phagocytosis and reduced leukocyte production in the bone marrow. In particular, progesterone triggers vasodilation in blood vessels and consequently increases endothelial permeability, managing to alter the function of periodontal ligament fibroblasts in collagen production, inhibiting the synthesis of collagen and non-collagen proteins, reducing folate levels, which promotes an imbalance in tissue repair [44, 45].

Several studies also show the relationship between sex hormones and changes in the production of inflammatory cytokines such as tumor necrosis factor, which has been quite associated with periodontal disease, as well as the IL-1 and IL-6, associated with bone resorption. In short, sex hormones promote a modulation of the host's response, such as increased vasodilation, vascular permeability, and inflammatory mediators, cytokines and prostaglandins, in the gingival tissue.

Although the studies have suitable clinical parameters to evaluate the use of contraceptives and also diagnose the periodontal diseases, the Fowkes and Fulton’s guideline was used, with the objective of qualifying the methods adopted in the studies, enabling the sufficiency assessment of these methods. The quality analysis indicated problems related to the sample size [19, 20, 22–25, 27, 28, 30–33, 36], suggesting the necessity of new studies with larger samples. The difficulties in carrying out population-based studies is well-known in the literature. Then, in the analytical perspective, at least the studies should include a large number of participants. The samples should be big enough for the probability of finding true statistically significant differences that demonstrate high clinical significance. However, this number should not be excessive, in order to avoid the waste of resources and the exposure of participants to unnecessary risk, for this in delineating these types of observational study it is necessary to have the presence of the realistic sample calculation [46].

In the quantitative analysis, it was sought to investigate the effect of hormonal contraceptives in CAL and PD clinical parameters. PD is a parameter that is associated with the inflammatory process. As inflammation increases, the probe penetrates more on the tissues. Moreover, CAL is related to the process of past periodontal destruction, in other words, how much periodontal tissue has already been lost as a result of inflammation. The combination of these two parameters is the gold standard for the evaluation of periodontal diseases. Recently, the new classification of periodontal diseases points to the necessity to exist a joint evaluation of these two parameters [47].

Despite the meta-analysis demonstrated the worst CAL in women users of contraceptives, PD data did not show a statistically significant difference. This information is amazing on the one hand, since lower rates of inflammation were observed. The possible explanation is because only one clinical examination was performed in most studies, without evaluation of longitudinality. On the other hand, the experience accumulated resulting from a persistent inflammatory process that was observed through the CAL with worse levels in the users of hormonal contraceptives. Furthermore, a few studies were included in the quantitative analysis, it could be contributed to this startling result. Currently, it is suggested that more parameters should be investigated to define the clinical picture of periodontitis. For severity, beyond CAL mensuration, the radiographic and quantification of tooth loss should be performed too. Moreover, for complexity, other parameters as furcation involvement, complex rehabilitation needs, the number of remaining teeth should be also analyzed.

Furthermore, the high heterogeneity presented among the included studies in meta-analysis indicated the presence of an impact in the validity of these results, this questioning was pointed in the certainty of the scientific evidence proposed by also the GRADE tool also.

The term “heterogeneity” refers to de dispersion of true effect across studies (I^2^) and the standard deviation between true effects (Tau^2^). In the random effect-model, the standard errors of the studies are adjusted to incorporate a measure of the extent variation (I^2^) among the the effect of the interventions observed in the studies (Tau^2^) [48]. It must take into account that the study effect varies according to characteristics of its population (age), interventions (hormonal dose), possible confounding factors (systemic diseases, oral hygiene habits, etc.), and other parameters. Therefore, the studies estimate different, but related, interventions effect and these factors influence the I^2^. So, in the meta-analysis of the present systematic review a random-effect model was adopted.

Some studies included in this systematic review and meta-analysis have some limitations such as the different methods that were used to assess periodontal disease and others did not report the composition of the drug and/or the dose/time used by the patients. Other important information is that the introduction of combined hormonal contraceptives with low dosage as the first pregnancy prevention option used in the last 30 years [49, 50]. These medicines also have high efficacy as compared to high dose, and have better tolerability and fewer side effects [50]. In the present systematic review, there is a variability of performance in these studies, from 1971 (El-Shary [26]) to the year 2018 (Smadi et al. [20]), the use of different types of doses of oral hormonal contraceptives possibly contributed to different results in the summarized analyzes.

These steroid hormones not only are responsible for physiologic changes in different phases of a woman life, but also act in different organ systems, including the oral cavity [51, 52]. The scientific literature complements that the periodontium is uncertain target tissue of these steroids’ hormones, however, the biological meaning of this association is further clarified by the presence of receptors of these hormones in the periodontal tissues [53]. Thus, estrogen and progesterone levels present in the contraceptive’s composition can modulate biological response [54]. Moreover, it is believed that the high level of these hormones acts in the vascular system, which can influence gingival inflammation [37]. Currently, the changes on general oral health in women using hormonal contraceptives were studied [55]. Ali et al. [11] corroborate with the outcomes found in this systematic review, that the changes in the periodontium are inconclusive.

In this sense, the monitoring and guidance by a professional in the dental field has great importance to control and treat the possible effects arising from the use of hormonal contraceptives in the oral cavity associated with the use of hormonal contraceptives. It is known that periodontal disease is associated with other comorbidities in the body, such as a reduction in the individual's immune response, making him more susceptible to other diseases [56]. In addition, improving the quality of life of women using hormonal contraceptives. Moreover, further studies are necessary to investigate the relationship of each type of pharmacological agent, dose, and time of administration to better elucidate this relation.

Despite the majority of the studies presents a low risk of bias individually, the summarized results pointed methodological failures that, if corrected in future studies, will allow better clinical and epidemiological evidence, for example, increase in sample size, an accomplishment of all clinical parameters pertinent to periodontitis, follow-up of patients over time, between others will be important for further investigation into this association.

## Conclusions

The results observed in this review indicate a potential effect of hormonal contraceptives in the periodontium, but not allowing solid conclusions, and still does not allow the adoption of clear preventive measures related to the use of hormonal contraceptives and periodontal diseases.

## Supplementary Information

**Additional file 1.** PRISMA Checklist.

**Additional file 2.** Search Strategies.

## Data Availability

The datasets analyzed during the current study are available from the corresponding author on reasonable request.

## Abbreviations

BOP: Bleeding on probing
CAL: Clinical attachment loss
CI: Confidence interval
GI: Gingival index
GRADE: Grading of Recommendations Assessment, Development, and Evaluation
PI: Plaque index
PRISMA: Preferred Reporting Items for Systematic Reviews and Meta-Analyses
PD: Probing depth
SMD: Standard mean difference
