# Supplementary material for: Is the use of contraceptives associated with periodontal diseases? A systematic review and meta-analyses
Source: BMC Womens Health. 2021 Feb 1;21:48. doi: 10.1186/s12905-021-01180-0 (PMC7852091; doi:10.1186/s12905-021-01180-0)
Supplement: Supplementary file 2 — Additional file 2. Search Strategies. [file 12905_2021_1180_MOESM2_ESM.docx]

**Suplemmentary Table 2.** Search strategies

| **Pubmed**  **(257)** | **#1 AND #2** |
| --- | --- |
|  | **#1**  (((((((((((((((((((((((((((((((((((((((((((((((((((((((((((Contraceptives, Oral, Hormonal[MeSH Terms]) OR Contraceptives, Oral, Hormonal[Title/Abstract]) OR Oral Contraceptives, Hormonal[Title/Abstract]) OR Contraceptives, Hormonal Oral[Title/Abstract]) OR Hormonal Oral Contraceptives[Title/Abstract]) OR Oral Contraceptive Agents, Hormonal[Title/Abstract]) OR Contraceptive Agents, Oral, Hormonal[Title/Abstract]) OR Hormonal Oral Contraceptive Agents[Title/Abstract]) OR Contraceptive Agents, Estrogen[Title/Abstract]) OR Agents, Estrogen Contraceptive[Title/Abstract]) OR Estrogen Contraceptive Agents[Title/Abstract]) OR Contraceptives, Oral[MeSH Terms]) OR Oral Contraceptives[Title/Abstract]) OR Oral Contraceptives, Phasic[Title/Abstract]) OR Contraceptives, Phasic Oral[Title/Abstract]) OR Phasic Oral Contraceptives[Title/Abstract]) OR Oral Contraceptives, Low-Dose[Title/Abstract]) OR Contraceptives, Low-Dose Oral[Title/Abstract]) OR Low-Dose Oral Contraceptives[Title/Abstract]) OR Oral Contraceptives, Low Dose[Title/Abstract]) OR Contraceptives, Oral[Title/Abstract]) OR Contraceptive Agents[MeSH Terms]) OR Contraceptive Agents[Title/Abstract]) OR Agents, Contraceptive[Title/Abstract]) OR Contraceptives[Title/Abstract]) OR Contraceptive Effect[Title/Abstract]) OR Effect, Contraceptive[Title/Abstract]) OR Contraceptive Effects[Title/Abstract]) OR Effects, Contraceptive[Title/Abstract]) OR Reproductive Control Agents[MeSH Terms]) OR Reproductive Control Agents[Title/Abstract]) OR Agents, Reproductive Control[Title/Abstract]) OR Control Agents, Reproductive[Title/Abstract]) OR Contraceptive Agents, Female[MeSH Terms]) OR Contraceptive Agents, Female[Title/Abstract]) OR Agents, Female Contraceptive[Title/Abstract]) OR Female Contraceptive Agents[Title/Abstract]) OR Female Contraceptive Agents[Title/Abstract]) OR Female Contraceptives[Title/Abstract]) OR Contraceptives, Female[Title/Abstract]) OR Vaccines, Contraceptive[MeSH Terms]) OR Vaccines, Contraceptive[Title/Abstract]) OR Vaccines, Antifertility[Title/Abstract]) OR Antifertility Vaccines[Title/Abstract]) OR Contraceptive Vaccines[Title/Abstract]) OR Contraceptives, Oral, Combined[MeSH Terms]) OR Contraceptives, Oral, Combined[Title/Abstract]) OR Contraceptive Agents, Female, Combined[Title/Abstract]) OR Oral Contraceptives, Combined[Title/Abstract]) OR Combined Oral Contraceptives[Title/Abstract]) OR Contraceptives, Combined Ora[Title/Abstract]) OR Contraceptives, Oral, Sequential[MeSH Terms]) OR Contraceptives, Oral, Sequential[Title/Abstract]) OR Oral Contraceptives, Sequential[Title/Abstract]) OR Contraceptives, Sequential Oral[Title/Abstract]) OR Sequential Oral Contraceptives[Title/Abstract]) OR Sequential Oral Contraceptive Agents[Title/Abstract]) OR Contraceptive Agents, Oral, Sequential[Title/Abstract]) OR Oral Contraceptive Agents, Sequential[Title/Abstract]) OR Contraceptive Agents, Female Sequential[Title/Abstract]  **# 2**   (((((((((((((((((((((((((((((((((((((((((((((((((((((((((((((((((((((((((((((((((((((((((Periodontal Attachment Loss[MeSH Terms]) OR Periodontal Attachment Loss[Title/Abstract]) OR Attachment Loss, Periodontal[Title/Abstract]) OR Loss, Periodontal Attachment[Title/Abstract]) OR Chronic Periodontitis[Title/Abstract]) OR Chronic Periodontitis[Title/Abstract]) OR Chronic Periodontitides[Title/Abstract]) OR Periodontitides, Chronic[Title/Abstract]) OR Periodontitis, Chronic[Title/Abstract]) OR Adult Periodontitis[Title/Abstract]) OR Adult Periodontitides[Title/Abstract]) OR Periodontitides, Adult[Title/Abstract]) OR Periodontitis, Adult[Title/Abstract]) OR Oral Health[MeSH Terms]) OR Oral Health[Title/Abstract]) OR Periodontal Diseases[MeSH Terms]) OR Periodontal Diseases[Title/Abstract]) OR Disease, Periodontal[Title/Abstract]) OR Diseases, Periodontal[Title/Abstract]) OR Periodontal Disease[Title/Abstract]) OR Parodontosis[Title/Abstract]) OR Parodontoses[Title/Abstract]) OR Pyorrhea Alveolaris[Title/Abstract]) OR Gingival Diseases[MeSH Terms]) OR Gingival Diseases[Title/Abstract]) OR Disease, Gingival[Title/Abstract]) OR Diseases, Gingival[Title/Abstract]) OR Gingival Disease[Title/Abstract]) OR Epulides[Title/Abstract]) OR Epulis[Title/Abstract]) OR Gingivosis[Title/Abstract]) OR Gingivoses[Title/Abstract]) OR Gingivitis[MeSH Terms]) OR Gingivitis[Title/Abstract]) OR Gingivitides[Title/Abstract]) OR Periodontium[MeSH Terms]) OR Periodontium[Title/Abstract]) OR Periodontiums[Title/Abstract]) OR Tooth Supporting Structures[Title/Abstract]) OR Structure, Tooth Supporting[Title/Abstract]) OR Structures, Tooth Supporting[Title/Abstract]) OR Supporting Structure, Tooth[Title/Abstract]) OR Supporting Structures, Tooth[Title/Abstract]) OR Tooth Supporting Structure[Title/Abstract]) OR Parodontium[Title/Abstract]) OR Parodontiums[Title/Abstract]) OR Paradentium[Title/Abstract]) OR Paradentiums[Title/Abstract]) OR Gingiva[MeSH Terms]) OR Gingiva[Title/Abstract]) OR Gums[Title/Abstract]) OR Gum[Title/Abstract]) OR Interdental Papilla[Title/Abstract]) OR Papilla, Interdental[Title/Abstract]) OR Tooth[MeSH Terms]) OR Tooth[Title/Abstract]) OR Teeth[Title/Abstract]) OR periodontitis[MeSH Terms]) OR periodontitis[Title/Abstract]) OR Periodontitides[Title/Abstract]) OR Pericementitis[Title/Abstract]) OR Pericementitides[Title/Abstract]) OR Alveolar Process[MeSH Terms]) OR Alveolar Process[Title/Abstract]) OR Alveolar Processes[Title/Abstract]) OR Process, Alveolar[Title/Abstract]) OR Processes, Alveolar[Title/Abstract]) OR Alveolar Ridge[Title/Abstract]) OR Ridge, Alveolar[Title/Abstract]) OR Alveolar Bone Loss[MeSH Terms]) OR Alveolar Bone Loss[Title/Abstract]) OR Alveolar Bone Losses[Title/Abstract]) OR Alveolar Process Atrophy[Title/Abstract]) OR Alveolar Process Atrophies[Title/Abstract]) OR Alveolar Resorption[Title/Abstract]) OR Alveolar Resorptions[Title/Abstract]) OR Resorption, Alveolar[Title/Abstract]) OR Resorptions, Alveolar[Title/Abstract]) OR Bone Loss, Periodontal[Title/Abstract]) OR Bone Losses, Periodontal[Title/Abstract]) OR Periodontal Bone Losses[Title/Abstract]) OR Periodontal Bone Loss[Title/Abstract]) OR Periodontal Resorption[Title/Abstract]) OR Periodontal Resorptions[Title/Abstract]) OR Resorption, Periodontal[Title/Abstract]) OR Alveolar Bone Atrophy[Title/Abstract]) OR Alveolar Bone Atrophies[Title/Abstract]) OR Bone Atrophies, Alveolar[Title/Abstract]) OR Bone Atrophy, Alveolar[Title/Abstract]) OR Bone Loss, Alveolar[Title/Abstract] |
| **Scopus**  **(663)** | **#1 AND #2** |
|  | **# 1** ( ( ( TITLE-ABS-KEY ( "Contraceptives, Oral, Hormonal" )  OR  TITLE-ABS-KEY ( "Oral Contraceptives, Hormonal" )  OR  TITLE-ABS-KEY ( "Contraceptives, Hormonal Oral" )  OR  TITLE-ABS-KEY ( "Hormonal Oral Contraceptives" )  OR  TITLE-ABS-KEY ( "Oral Contraceptive Agents, Hormonal" )  OR  TITLE-ABS-KEY ( "Contraceptive Agents, Oral, Hormonal" )  OR  TITLE-ABS-KEY ( "Hormonal Oral Contraceptive Agents" )  OR  TITLE-ABS-KEY ( "Contraceptive Agents, Estrogen" )  OR  TITLE-ABS-KEY ( "Agents, Estrogen Contraceptive" )  OR  TITLE-ABS-KEY ( "Estrogen Contraceptive Agents" )  OR  TITLE-ABS-KEY ( "Contraceptives, Oral" )  OR  TITLE-ABS-KEY ( "Oral Contraceptives" )  OR  TITLE-ABS-KEY ( "Oral Contraceptives, Phasic" )  OR  TITLE-ABS-KEY ( "Contraceptives, Phasic Oral" )  OR  TITLE-ABS-KEY ( "Phasic Oral Contraceptives" )  OR  TITLE-ABS-KEY ( "Oral Contraceptives, Low-Dose" )  OR  TITLE-ABS-KEY ( "Contraceptives, Low-Dose Oral" )  OR  TITLE-ABS-KEY ( "Low-Dose Oral Contraceptives" )  OR  TITLE-ABS-KEY ( "Oral Contraceptives, Low Dose" ) ) )  OR  ( ( TITLE-ABS-KEY ( "Contraceptive Agents" )  OR  TITLE-ABS-KEY ( "Agents,Contraceptive" )  OR  TITLE-ABS-KEY ( "Contraceptives" )  OR  TITLE-ABS-KEY ( "Contraceptive Effect" )  OR  TITLE-ABS-KEY ( "Effect,Contraceptive" )  OR  TITLE-ABS-KEY ( "Contraceptive Effects" )  OR  TITLE-ABS-KEY ( "Effects,Contraceptive" )  OR  TITLE-ABS-KEY ( "Reproductive Control Agents" )  OR  TITLE-ABS-KEY ( "Agents,Reproductive Control" )  OR  TITLE-ABS-KEY ( "Control Agents,Reproductive" )  OR  TITLE-ABS-KEY ( "Contraceptive Agents,Female" )  OR  TITLE-ABS-KEY ( "Agents,Female Contraceptive" )  OR  TITLE-ABS-KEY ( "Female Contraceptive Agents" )  OR  TITLE-ABS-KEY ( "Contraceptives,Female" )  OR  TITLE-ABS-KEY ( "Female Contraceptives" )  OR  TITLE-ABS-KEY ( "Vaccines,Contraceptive" )  OR  TITLE-ABS-KEY ( "Vaccines,Antifertility" )  OR  TITLE-ABS-KEY ( "Antifertility Vaccines" )  OR  TITLE-ABS-KEY ( "Contraceptive Vaccines" ) ) )  OR  ( ( TITLE-ABS-KEY ( "Contraceptives, Oral, Combined" )  OR  TITLE-ABS-KEY ( "Contraceptive Agents, Female, Combined" )  OR  TITLE-ABS-KEY ( "Oral Contraceptives,Combined" )  OR  TITLE-ABS-KEY ( "Combined Oral Contraceptives" )  OR  TITLE-ABS-KEY ( "Contraceptives,Combined Oral" )  OR  TITLE-ABS-KEY ( "Contraceptives, Oral, Sequential" )  OR  TITLE-ABS-KEY ( "Oral Contraceptives,Sequential" )  OR  TITLE-ABS-KEY ( "Contraceptives,Sequential Oral" )  OR  TITLE-ABS-KEY ( "Sequential Oral Contraceptives" )  OR  TITLE-ABS-KEY ( "Sequential Oral Contraceptive Agents" )  OR  TITLE-ABS-KEY ( "Contraceptive Agents, Oral, Sequential" )  OR  TITLE-ABS-KEY ( "Oral Contraceptive Agents, Sequential" )  OR  TITLE-ABS-KEY ( "Contraceptive Agents, Female Sequential" ) ) ) ) **#2** ( ( ( TITLE-ABS-KEY ( "Periodontal Attachment Loss" )  OR  TITLE-ABS-KEY ( "Attachment Loss, Periodontal" )  OR  TITLE-ABS-KEY ( "Loss, Periodontal Attachment" )  OR  TITLE-ABS-KEY ( "Chronic Periodontitis" )  OR  TITLE-ABS-KEY ( "Chronic Periodontitides" )  OR  TITLE-ABS-KEY ( "Periodontitides, Chronic" )  OR  TITLE-ABS-KEY ( "Periodontitis, Chronic" )  OR  TITLE-ABS-KEY ( "Adult Periodontitis" )  OR  TITLE-ABS-KEY ( "Adult Periodontitides" )  OR  TITLE-ABS-KEY ( "Periodontitides, Adult" )  OR  TITLE-ABS-KEY ( "Periodontitis, Adult" )  OR  TITLE-ABS-KEY ( "Oral Health" )  OR  TITLE-ABS-KEY ( "Periodontal Diseases" )  OR  TITLE-ABS-KEY ( "Disease, Periodontal" )  OR  TITLE-ABS-KEY ( "Diseases, Periodontal" )  OR  TITLE-ABS-KEY ( "Periodontal Disease" )  OR  TITLE-ABS-KEY ( parodontosis )  OR  TITLE-ABS-KEY ( parodontoses )  OR  TITLE-ABS-KEY ( "Pyorrhea Alveolaris" ) ) )  OR  ( ( TITLE-ABS-KEY ( "Gingival Diseases" )  OR  TITLE-ABS-KEY ( "Disease, Gingival" )  OR  TITLE-ABS-KEY ( "Diseases, Gingival" )  OR  TITLE-ABS-KEY ( "Gingival Disease" )  OR  TITLE-ABS-KEY ( epulides )  OR  TITLE-ABS-KEY ( epulis )  OR  TITLE-ABS-KEY ( gingivosis )  OR  TITLE-ABS-KEY ( gingivoses )  OR  TITLE-ABS-KEY ( gingivitis )  OR  TITLE-ABS-KEY ( gingivitides )  OR  TITLE-ABS-KEY ( periodontium )  OR  TITLE-ABS-KEY ( periodontiums )  OR  TITLE-ABS-KEY ( "Tooth Supporting Structures" )  OR  TITLE-ABS-KEY ( "Structure, Tooth Supporting" )  OR  TITLE-ABS-KEY ( "Structures, Tooth Supporting" ) ) )  OR  ( ( TITLE-ABS-KEY ( parodontium )  OR  TITLE-ABS-KEY ( parodontiums )  OR  TITLE-ABS-KEY ( paradentium )  OR  TITLE-ABS-KEY ( paradentiums )  OR  TITLE-ABS-KEY ( gingiva )  OR  TITLE-ABS-KEY ( gums )  OR  TITLE-ABS-KEY ( gum )  OR  TITLE-ABS-KEY ( "Interdental Papilla" )  OR  TITLE-ABS-KEY ( "Papilla, Interdental" )  OR  TITLE-ABS-KEY ( tooth )  OR  TITLE-ABS-KEY ( teeth )  OR  TITLE-ABS-KEY ( periodontitis )  OR  TITLE-ABS-KEY ( periodontitides )  OR  TITLE-ABS-KEY ( pericementitis )  OR  TITLE-ABS-KEY ( pericementitides ) ) )  OR  ( ( TITLE-ABS-KEY ( "Alveolar Process" )  OR  TITLE-ABS-KEY ( "Alveolar Processes" )  OR  TITLE-ABS-KEY ( "Alveolar Processes" )  OR  TITLE-ABS-KEY ( "Process, Alveolar" )  OR  TITLE-ABS-KEY ( "Processes, Alveolar" )  OR  TITLE-ABS-KEY ( "Alveolar Ridge" )  OR  TITLE-ABS-KEY ( "Ridge, Alveolar" )  OR  TITLE-ABS-KEY ( "Alveolar Bone Loss" )  OR  TITLE-ABS-KEY ( "Alveolar Bone Losses" )  OR  TITLE-ABS-KEY ( "Alveolar Process Atrophy" )  OR  TITLE-ABS-KEY ( "Alveolar Process Atrophies" )  OR  TITLE-ABS-KEY ( "Alveolar Resorption" )  OR  TITLE-ABS-KEY ( "Alveolar Resorptions" )  OR  TITLE-ABS-KEY ( "Resorption, Alveolar" )  OR  TITLE-ABS-KEY ( "Resorptions, Alveolar" )  OR  TITLE-ABS-KEY ( "Bone Loss, Periodontal" )  OR  TITLE-ABS-KEY ( "Bone Losses, Periodontal" )  OR  TITLE-ABS-KEY ( "Periodontal Bone Losses" )  OR  TITLE-ABS-KEY ( "Periodontal Bone Loss" )  OR  TITLE-ABS-KEY ( "Periodontal Resorption" )  OR  TITLE-ABS-KEY ( "Periodontal Resorptions" )  OR  TITLE-ABS-KEY ( "Resorption, Periodontal" )  OR  TITLE-ABS-KEY ( "Alveolar Bone Atrophy" )  OR  TITLE-ABS-KEY ( "Alveolar Bone Atrophies" )  OR  TITLE-ABS-KEY ( "Bone Atrophies, Alveolar" )  OR  TITLE-ABS-KEY ( "Bone Atrophy, Alveolar" )  OR  TITLE-ABS-KEY ( "Bone Loss, Alveolar" ) ) ) ) |
| **Web of Science**  **(74)** | **#1 AND #2** |
|  | **#1**  "Contraceptives, Oral, Hormonal") *OR* **TOPIC:** ("Oral Contraceptives, Hormonal") *OR* **TOPIC:**("Contraceptives, Hormonal Oral") *OR* **TOPIC:** ("Hormonal Oral Contraceptives") *OR* **TOPIC:** ("Oral Contraceptive Agents, Hormonal") *OR* **TOPIC:** ("Contraceptive Agents, Oral, Hormonal") *OR* **TOPIC:** ("Hormonal Oral Contraceptive Agents") *OR* **TOPIC:** ("Contraceptive Agents, Estrogen") *OR* **TOPIC:** ("Agents, Estrogen Contraceptive") *OR* **TOPIC:** ("Estrogen Contraceptive Agents") *OR* **TOPIC:** ("Contraceptive Agents") *OR* **TOPIC:**("Agents, Contraceptive") *OR* **TOPIC:** ("Contraceptive Effect*") *OR* **TOPIC:** ("Effect*, Contraceptive")  **#2:**   ("Contraceptive Agents, Female")*OR* **TOPIC:**("Agents, Female Contraceptive")*OR***TOPIC:**("Female Contraceptive Agents") *OR* **TOPIC:**("Contraceptives, Female") *OR* **TOPIC:**("Female Contraceptives") *OR* **TOPIC:**("Contraceptives, Oral") *OR* **TOPIC:**("Oral Contraceptives") *OR* **TOPIC:**("Oral Contraceptives, Phasic") *OR***TOPIC:**("Contraceptives, Phasic Oral") *OR* **TOPIC:**("Oral Contraceptives, Low-Dose") *OR* **TOPIC:**("Contraceptives, Low-Dose Oral") *OR* **TOPIC:**("Low-Dose Oral Contraceptives") *OR* **TOPIC:**("Oral Contraceptives, Low Dose")  **#3**  ("Contraceptives, Oral, Combined") *OR* **TOPIC:**("Contraceptive Agents, Female, Combined") *OR* **TOPIC:**("Oral Contraceptives, Combined") *OR* **TOPIC:**("Combined Oral Contraceptives") *OR* **TOPIC:**("Contraceptives, Combined Oral") *OR* **TOPIC:**("Contraceptives, Oral, Sequentiall") *OR* **TOPIC:**("Oral Contraceptives, Sequential") *OR* **TOPIC:**("Contraceptives, Sequential Oral") *OR* **TOPIC:**("Sequential Oral Contraceptive Agents") *OR* **TOPIC:**("Contraceptive Agents, Oral, Sequential") *OR* **TOPIC:**("Oral Contraceptive Agents, Sequential") *OR* **TOPIC:**("Vaccines, Contraceptive") *OR* **TOPIC:**("Vaccines, Antifertility") *OR* **TOPIC:**("Antifertility Vaccines") *OR* **TOPIC:**("Contraceptive Vaccines") *OR* **TOPIC:**("Reproductive Control Agents") *OR* **TOPIC:**("Agents, Reproductive Control") *OR* **TOPIC:**("Control Agents, Reproductive")  **#1 OR #2 OR #3 = #4**  **#5**  ("PeriodontalDisease*") *OR* **TOPIC:** ("Disease*,Periodontal") *OR* **TOPIC:** ("Parodontosis") *OR* **TOPIC:**("Parodontoses") *OR* **TOPIC:** ("PyorrheaAlveolaris") *OR* **TOPIC:** ("GingivalDisease*") *OR* **TOPIC:** ("Disease*,Gingival") *OR* **TOPIC:** (Epulides) *OR* **TOPIC:** (Epulis) *OR* **TOPIC:** (Gingivosis) *OR* **TOPIC:** (Gingivitis) *OR* **TOPIC:**(Gingivitides) *OR* **TOPIC:** (Periodontium) *OR* **TOPIC:** (Periodontiums) *OR* **TOPIC:** ("Tooth Supporting Structure*") *OR* **TOPIC:** ("Structure*, Tooth Supporting") *OR* **TOPIC:** ("Supporting Structure*, Tooth") *OR* **TOPIC:** (Parodontium*) *OR* **TOPIC:** (Paradentium*)  **#6**  (Gingiva) *OR***TOPIC:** (Gum*) *OR* **TOPIC:** ("interdentalPapilla") *OR***TOPIC:** ("Papilla,Interdental") *OR***TOPIC:** (tooth) *OR*  **TOPIC:** (Teeth) *OR*  **TOPIC:** ("Alveolar Process*") *OR* **TOPIC:** ("Process, Alveolar") *OR*  **TOPIC:** ("Alveolar Ridge") *OR* **TOPIC:** ("Ridge,Alveolar") *OR*  **TOPIC:** (Periodontitis) *OR*  **TOPIC:** (Periodontitides) *OR*  **TOPIC:** (Pericementitis) *OR* **TOPIC:**(Pericementitides) *OR* **TOPIC:** ("Chronic Periodontitis") *OR*  **TOPIC**("Chronic Periodontitides") *OR*  **TOPIC** ("Periodontitides, Chronic") *OR*  **TOPIC** ("Periodontitis, Chronic") *OR*  **TOPIC** Adult Periodontitis") *OR*  **TOPIC** ("Adult Periodontitides") *OR*  **TOPIC** ("Periodontitides, Adult") *OR*  **TOPIC** ("Periodontitis, Adult”)  **#7**  ("Alveolar Bone Loss*") *OR*  **TOPIC** ("Alveolar Process Atrophy") *OR*  **TOPIC:** ("Alveolar Process Atrophies") *OR*  **TOPIC:** ("Alveolar Resorption*") *OR*  **TOPIC** ("Resorption*, Alveolar") *OR*  **TOPIC:** (Teeth) *OR*  **TOPIC:** ("Alveolar Process*") *OR*  **TOPIC:** ("Process, Alveolar") *OR*  **TOPIC** ("Bone Loss*, Periodontal") *OR*  **TOPIC:** ("Periodontal Bone Loss*") *OR*  **TOPIC:**("Periodontal Resorption*") *OR*  **TOPIC** ("Resorption, Periodontal") *OR*  **TOPIC** ("Alveolar Bone Atrophy") *OR*  **TOPIC** ("Alveolar Bone Atrophies") *OR*  **TOPIC** ("Bone Atrophies, Alveolar") *OR*  **TOPIC** ("Bone Loss, Alveolar") *OR*  **TOPIC** ("Oral Health")  **TOPIC** ("Health, Oral")  **#5 OR #6 OR #7 = #8**  **#8 AND #4** |
| **Lilacs**  **(225)** | **#1 AND #2** |
|  | **#1-**  Contraceptives, Oral, Hormonal OR Oral Contraceptives, Hormonal, Contraceptives, Hormonal Oral OR Hormonal Oral Contraceptives OR Oral Contraceptive Agents, Hormonal OR Contraceptive Agents, Oral, Hormonal OR Hormonal Oral Contraceptive Agents OR Contraceptive Agents, Estrogen OR Agents, Estrogen Contraceptive OR Estrogen Contraceptive Agents OR Contraceptive Agents OR Agents, Female Contraceptive OR Female Contraceptive Agents OR Contraceptives, Female OR Female Contraceptivs OR Contraceptives, Oral OR Oral Contraceptives OR Oral Contraceptives, Phasic OR Contraceptives, Phasic Oral OR Phasic Oral Contraceptives OR Oral Contraceptives, Low-Dose OR Contraceptives, Low-Dose Oral OR Low-Dose Oral Contraceptives OR Oral Contraceptives, Low Dose OR Contraceptives, Oral, Combined OR Contraceptive Agents, Female, Combined OR Oral Contraceptives, Combined OR Combined Oral Contraceptives OR Contraceptives, Combined Oral OR Contraceptives, Oral, Sequential OR Sequential Oral Contraceptives OR Sequential Oral Contraceptive Agents OR Contraceptive Agents, Oral, Sequential OR Oral Contraceptive Agents, Sequential) OR Contraceptive Agents, Female Sequential OR Vaccines, Contraceptive OR Vaccines, Antifertility OR Antifertility Vaccines OR Contraceptive Vaccines OR Reproductive Control Agents OR Agents, Reproductive Control OR Control Agents, Reproductive  **#2-**  Periodontal Diseases OR Diseases, Periodontal OR Paradontosis OR Paradontoses OR Pyorrhea Alveolaris OR Gingival Diseases OR Diseases, Gingival OR Epulides OR Epulis OR Gingivosis OR Gingivitis OR Gingivitides OR Periodontium OR Tooth Supporting Structure OR Structure, Tooth Supporting OR Supporting Structure, Tooth OR Parodontium OR Paradentium OR Alveolar Process OR Process, Alveolar OR Alveolar Ridge OR Ridge, Alveolar OR Periodontitis OR Periodontitides OR Pericementitis OR Chronic Periodontitis OR Chronic Periodontitides OR Periodontitides, Chronic OR Adult Periodontitis OR Adult Periodontitides OR Periodontitides, Adult OR Periodontitis, Adult OR Periodontal Attachment Loss OR Attachment Loss, Periodontal OR Loss, Periodontal Attachment OR Alveolar Bone Loss OR Alveolar Process Atrophy OR Alveolar Process Atrophies OR Alveolar Resorption OR Resorption, Alveolar OR Bone Loss, Periodontal OR Periodontal Bone Loss OR Periodontal Resorption OR Resorption, Periodontal OR Alveolar Bone Atroph OR Bone Atroph, Alveolar OR Bone Loss, Alveolar |
| **Cochrane**  **(7)** | #1 AND #2 |
|  | #1 ("Contraceptives, Oral, Hormonal"):ti,ab,kw OR ("Contraceptives, Oral"):ti,ab,kw OR ("Contraceptive Agents"):ti,ab,kw OR ("Vaccines, Contraceptive"):ti,ab,kw OR ("Reproductive Control Agents"):ti,ab,kw"'#2("Chronic Periodontitis"):ti,ab,kw OR ("Periodontal Diseases"):ti,ab,kw OR (Gingivitis):ti,ab,kw OR (periodontitis):ti,ab,kw OR ("Alveolar Process"):ti,ab,kw"' |
| **Open Grey**  **(0)** | Contraceptives+Periodontitis |
| **Google Scholar**  **(5)** | "Contraceptives, Oral, Hormonal"+periodontitis |
| **Clinical Trials**  **(0)** | Contraceptives+Periodontitis |
